# Highly thermostable GH39 β-xylosidase from a *Geobacillus* sp. strain WSUCF1

**DOI:** 10.1186/s12896-014-0106-8

**Published:** 2014-12-23

**Authors:** Aditya Bhalla, Kenneth M Bischoff, Rajesh K Sani

**Affiliations:** Department of Chemical and Biological Engineering, South Dakota School of Mines and Technology, Rapid City, SD 57701 USA; Renewable Product Technology Research Unit, Agricultural Research Service, National Center for Agricultural Utilization Research, U.S. Department of Agriculture, Peoria, IL 61604 USA; Present address: Great Lakes Bioenergy Research Center, Michigan State University, East Lansing, MI 48824 USA

**Keywords:** Lignocellulose, Biofuels, β-xylosidase, Thermostable

## Abstract

**Background:**

Complete enzymatic hydrolysis of xylan to xylose requires the action of endoxylanase and β-xylosidase. β-xylosidases play an important part in hydrolyzing xylo-oligosaccharides to xylose. Thermostable β-xylosidases have been a focus of attention as industrially important enzymes due to their long shelf life and role in the relief of end-product inhibition of xylanases caused by xylo-oligosaccharides. Therefore, a highly thermostable β-xylosidase with high specific activity has significant potential in lignocellulose bioconversion.

**Results:**

A gene encoding a highly thermostable GH39 β-xylosidase was cloned from *Geobacillus* sp. strain WSUCF1 and expressed in *Escherichia coli*. Recombinant β-xylosidase was active over a wide range of temperatures and pH with optimum temperature of 70°C and pH 6.5. It exhibited very high thermostability, retaining 50% activity at 70°C after 9 days. WSUCF1 β-xylosidase is more thermostable than β-xylosidases reported from other thermophiles (growth temperature ≤ 70°C). Specific activity was 133 U/mg when incubated with p-nitrophenyl xylopyranoside, with K_m_ and V_max_ values of 2.38 mM and 147 U/mg, respectively. SDS-PAGE analysis indicated that the recombinant enzyme had a mass of 58 kDa, but omitting heating prior to electrophoresis increased the apparent mass to 230 kDa, suggesting the enzyme exists as a tetramer. Enzyme exhibited high tolerance to xylose, retained approximately 70% of relative activity at 210 mM xylose concentration. Thin layer chromatography showed that the enzyme had potential to convert xylo-oligomers (xylobiose, triose, tetraose, and pentaose) into fermentable xylose. WSUCF1 β-xylosidase along with WSUCF1 endo-xylanase synergistically converted the xylan into fermentable xylose with more than 90% conversion.

**Conclusions:**

Properties of the WSUCF1 β-xylosidase i.e. high tolerance to elevated temperatures, high specific activity, conversion of xylo-oligomers to xylose, and resistance to inhibition from xylose, make this enzyme potentially suitable for various biotechnological applications.

## Background

Xylan represents the second most abundant polysaccharide in the biosphere after cellulose, and therefore is a potential source of sugars for the production of biofuels. Complete degradation of xylan to monomeric sugars requires synergistic action of several xylanolytic enzymes. Endo-1,4-β-xylanases (EC 3.2.1.8) hydrolyze the xylan backbone and β-D-xylosidases (EC 3.2.1.37) sunder the resulting xylooligomers to free xylose [1,2]. Availability of free xylose is immensely important to the overall efficiency of conversion of biomass to biofuels in the fermentation industry.

Xylanases have also been suggested to complement the activity of cellulases for the efficient hydrolysis of lignocellulose [2,3]. Kumar and Wyman [4] reported increased efficiency of cellulases on lignocellulosic biomass after addition of endo-xylanase and β-xylosidase. β-xylosidase degrades xylose oligomers before or during enzymatic hydrolysis of lignocellulosic biomass to reduce inhibition on cellulases by xylose oligomers. But under industrial processes, xylose could accumulate to levels that strongly inhibit performance of the catalyst. To overcome this limitation, a xylose-tolerant xylosidase is desirable which can resist high concentrations of the xylose [5,6]. A number of thermostable β-xylosidases are reported from thermophilic and hyperthermophilic bacteria including species of the genera *Geobacillus*, *Thermoanaerobacter*, *Thermatoga*, and *Thermoanaerobacterium* [5,7-11]. The thermostable enzymes are of interest in industrial processes due to their long shelf lives, compatibility with heat pretreatment, decreased microbial contamination at high temperatures and activity for longer durations [12].

The bacterial β-D-xylosidases mainly belong to glycosyl hydrolase families GH 3, 30, 39, 43, 52, and 54. Family 39 enzymes belong to the largest glycoside hydrolase clan (GH-A), whose members share a TIM-barrel (β/α)_8_ structure and a conserved catalytic machinery [13]. Reports on characterization of GH39 family β-xylosidases from thermophilic microorganisms are scarce. In this study, we report the cloning and characterization of GH39 thermostable β-xylosidase from *Geobacillus* sp. strain WSUCF1. This strain was isolated from samples collected from a compost facility and is an aerobic spore forming thermophile [14].

## Results

### Phylogenetic analysis of WSUCF1 β-xylosidase

Genome sequence of WSUCF1 revealed several genes encoding glycoside hydrolases. Out of 865 ORFs for carbohydrate metabolism, 70 ORFs were found to be involved in polysaccharide degradation [15]. It was found that the WSUCF1 genome contained two genes coding for putative β-xylosidases - WP_020755811 (1509 bp) and WP_020755806 (2157 bp). The deduced amino acid sequence analysis of these two β-xylosidases using Pfam showed homology with that of the GH39 and GH52 family, respectively. Literature shows that there is only one report [8] on the cloning and characterization of a GH39 family β-xylosidase from *Geobacillus* spp. Therefore, the WP_020755811 β-xylosidase was selected for further study.

**Figure 1:**
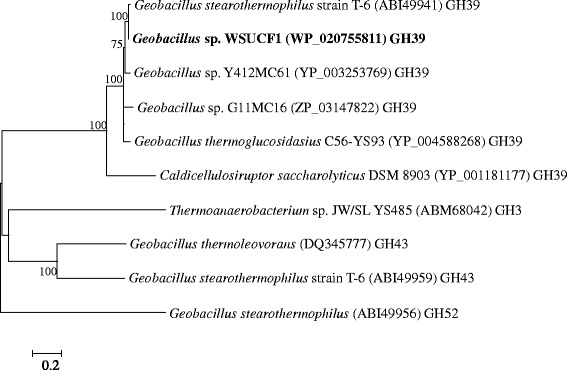
**Phylogenetic tree showing relationship between β-xylosidase sequence of** ***G*** **. sp. strain WSUCF1 and reference β-xylosidase sequences in GenBank, constructed using neighbor-joining method.** Bootstrap values which were ≥75% are indicated at the nodes. The scale bar represents 0.2 substitutions per amino acid position. Short title: Phylogenetic relationship between WSUCF1 β-xylosidase and reference β-xylosidase sequences.

Figure 1 shows the phylogenetic comparison of the putative WSUCF1 β-xylosidase amino acid sequence to other β-xylosidases. It shows that the WSUCF1 enzyme clusters with other GH39 family β-xylosidases. Within the GH39 group, β-xylosidase from WSUCF1 formed sister clades supported by high bootstrap values with sequences belonging to *Geobacillus* spp. (ABI49941, YP_003253769, and ZP_03147822). β-xylosidases from different GH families (e.g., GH3, GH43, and GH52) made separate clusters on the phylogenetic tree (Figure 1).

Alignment of the amino acid sequences of WSUCF1 β-xylosidase (WP_020755811) revealed 98, 95, and 91% identity with GH39 β-xylosidases from *Geobacillus stearothermophilus* strain T-6 (ABI49941), *Geobacillus* sp. Y412MC61 (YP_003253769), and *Geobacillus* sp. G11MC16 (ZP_03147822), respectively. β-xylosidase from *Geobacillus thermoleovorans* (DQ345777) belonging to GH43 family and *Geobacillus stearothermophilus* (ABI49956) belonging to GH52 family showed only 29 and 33% identity respectively.

### Expression, purification, and characterization of WSUCF1 β-xylosidase

**Figure 2:**
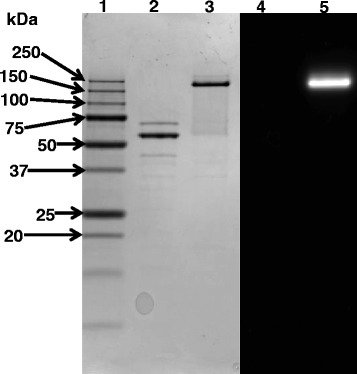
**SDS-PAGE (8-16% gradient) and zymogram of WSUCF1 β-xylosidase.** Lane 1, Precision Plus Protein Standards (BioRad); Lane 2, β-xylosidase with heat treatment (95°C for 2 min); Lane 3, β-xylosidase without any heat treatment; Lane 4, activity staining of β-xylosidase with heat treatment; and Lane 5, activity staining of β-xylosidase without any heat treatment. Short title: SDS-PAGE and zymogram of WSUCF1 β-xylosidase.

For functional analysis, the gene encoding WP_020755811 β-xylosidase was amplified from the *Geobacillus* sp. strain WSUCF1 genome. The gene was cloned in the pRham™ N-His SUMO Kan vector, expressed in *E. coli,* and purified using Ni-NTA affinity chromatography. SDS-PAGE analysis of the purified β-xylosidase showed a prominent band of 58 kDa (Figure 2, Lane 2) which was consistent with the predicted mass of the β-xylosidase enzyme. No enzyme activity was detected by zymogram analysis of the 58 kDa protein. By omitting the heat treatment prior to electrophoresis, the apparent mass of the prominent protein increased to 230 kDa, which displayed β-xylosidase activity in zymogram analysis (Figure 2, Lanes 3 and 5).

**Figure 3:**
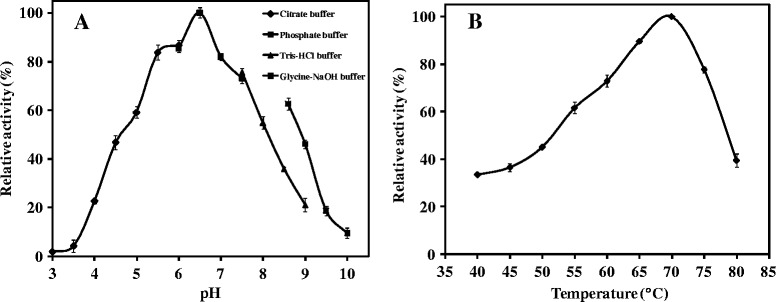
**Impact of pH (A) and temperature (B) on the WSUCF1 β-xylosidase activity.** The enzyme activities are expressed as percentages of the maximum activity (125.2 U/mg). The points are the averages of triplicates, and error bars indicate ± standard deviations of the means (n = 3). Error bars smaller than the symbols are not shown. Short title: Impact of pH **(A)** and temperature **(B)** on the WSUCF1 β-xylosidase activity.

The recombinant β-xylosidase from WSUCF1 exhibited activity in a broad range of pH (4.0-9.5) with optima at 6.5 (Figure 3A). β-xylosidase activity increased linearly when pH was increased from 4 to 6.5, but decreased when pH was further increased from 6.5 to 10. It retained more than 80% activity in the pH range of 5.5 - 7. At high pH of 8.6 and 9 (glycine- NaOH buffer), it retained 62 and 46% relative activity respectively. Effect of different temperatures on WSUCF1 xylosidase activity is shown in Figure 3B. It exhibited maximum activity at temperature of 70°C. The enzyme was active between temperatures of 50 to 75°C, with more than 50% relative activity.

**Figure 4:**
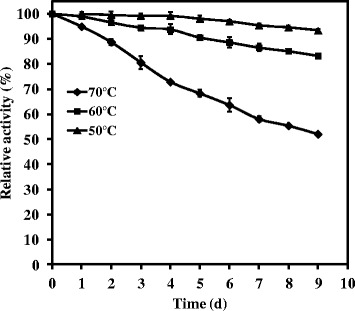
**Thermal stability of WSUCF1 β-xylosidase.** Enzyme activities are expressed as percentages of the initial activity (100%). The points are the averages of triplicates, and error bars indicate ± standard deviations of the means (n = 3). Error bars smaller than the symbols are not shown. Short title: Thermal stability of WSUCF1 β-xylosidase.

WSUCF1 β-xylosidase was highly thermostable in the range of 50-70°C (Figure 4). The enzyme was optimally stable at 50°C for 1 day without any loss of activity while about 93% activity was retained after incubation for 9 days. At 60°C, about 99% residual activity was detectable after 1 day of incubation and it lost only 17% of its original activity after incubation for 9 days. The enzyme retained 94% of its original activity after incubation of 1 day at 70°C, with a half-life of 9 days. The effect of the presence of metal ions on enzyme activity was studied (Table 1). WSUCF1 β-xylosidase retained its activity in the presence of different metal ions except Cu^2+^ and Hg^2+^. More than 70% activity was retained in the presence of Zn^2+^, Co^2+^, Ni^2+^, Ca^2+^, Mn^2+^, and Mg^2+^ whereas it lost 73 and 100% activity in the presence of Cu^2+^ and Hg ^2+^, respectively.

**Table 1:** **Effect of different metal ions on β-xylosidase activity**

**Metal ions**	**Relative activity (%)**
Control	100.00 ± 2.69
Mn^2+^	101.18 ± 2.32
Ca^2+^	96.04 ± 0.95
Mg^2+^	91.33 ± 2.97
Co^2+^	84.69 ± 3.20
Ni^2+^	73.17 ± 2.95
Cu^2+^	27.79 ± 1.23
Zn^2+^	74.71 ± 2.37
Hg^2+^	0

The kinetic parameters were calculated from Lineweaver-Burke plots of specific activities at various substrate concentrations (data not shown). The K_m_ and V_max_ values for WSUCF1 β-xylosidase with paranitrophenyl- beta-xylopyranoside (pNP-X) were 2.38 mM and 147.0 U/mg, respectively. The substrate specificity of the purified WSUCF1 β-xylosidase was determined using various substrates. WSUCF1 β-xylosidase was highly active on p-nitrophenyl β-D-xylopyranoside (pNPX). No specificity was observed for other p-NP substrates such as p-nitrophenyl β-D-cellobioside, p-nitrophenyl α-D-xylopyranoside, p-nitrophenyl α-L-arabinofuranoside, and p-nitrophenyl β-D-glucopyranoside (data not shown). In addition, polysaccharides such as oat-spelt xylan, beechwood xylan, and birchwood xylan did not serve as substrates for WSUCF1 β-xylosidase.

**Figure 5:**
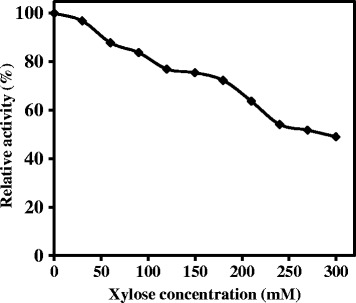
**Effect of different concentrations of xylose on WSUCF1 β-xylosidase activity.** The enzyme activities are expressed as percentages of the maximum activity (143.4 U/mg). The points are the averages of duplicates, and error bars indicate standard deviations of the means (n = 3). Short title: Effect of xylose on WSUCF1 β-xylosidase activity.

Effect of various xylose concentrations on WSUCF1 β-xylosidase activity was investigated. Results showed that concentrations of up to 50 mM xylose did not have much inhibitory effect. The enzyme retained 85 and 66% of its activity with 50 and 210 mM xylose concentrations, respectively (Figure 5). When the xylose concentration was increased to 300 mM, the enzyme still retained 50% activity. These data suggest that the WSUCF1 β-xylosidase is resistant to inhibition from high concentrations of xylose.

### Hydrolysis of xylo-oligosaccharides and xylan

**Figure 6:**
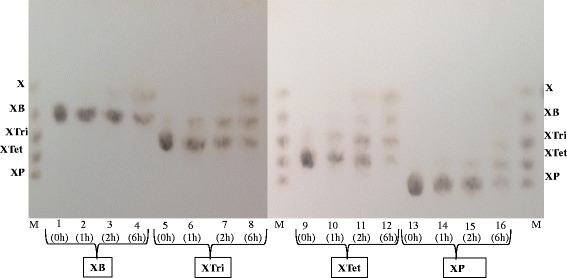
**Thin layer chromatography of hydrolysis of xylooligosaccharides.** Lane M, mixture of xylooligosaccharides (xylose (X), xylobiose (XB), xylotriose (XTri) and xylotetraose (Xtet), xylopentaose (XP) (1.5% each, wt/vol). Lane 1, Lane 5, Lane 9, Lane 13 - XB, XT, XTet, XP, respectively (Controls without enzyme); Lane 2–4, Lane 6–8, Lane 10–12, Lane 14–16 - XB, XT, XTet, XP (4 μl, 1.5%, wt/vol) each sugar was incubated with WSUCF1 β-xylosidase (0.05 U) for 1 h, 2 h, 6 h respectively. Short title: Thin layer chromatography of hydrolysis of xylooligosaccharides.

Thin layer chromatography (TLC) results showed that WSUCF1 β-xylosidase actively hydrolyzed xylo-oligosaccharides (Figure 6). It was found that enzyme was active on all xylo-oligosaccharides and released xylose and the other xylo-oligosaccharides with a degree of polymerization1 unit smaller than the substrate. For example, after 2 hours of hydrolysis, most of the xylotetraose was converted into smaller units with increase in xylose concentration. In case of xylopentaose hydrolysis, after 6 hours xylotetraose, xylotriose, xylobiose, and xylose were detected. With increase in time of hydrolysis of xylo-oligosaccharides from 2 to 6 hours, xylose yields were increased (Figure 6).

**Figure 7:**
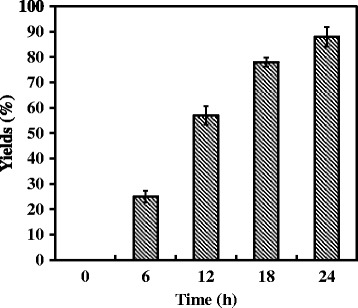
**Synergistic action of endoxylanase and β-xylosidase on hydrolysis of xylan.** Twenty mg xylan was incubated with WSUCF1 endoxylanase (5 mg/g xylan) and β-xylosidase (2.5 mg/g xylan) at 60°C for 24 h. Yields (100%) is equal to the theoretical maximum concentration of xylose released from xylan. Short title: Synergistic action of endoxylanase and β-xylosidase on xylan hydrolysis.

Mixtures of recombinant WSUCF1 endo-xylanase [16] and WSUCF1 β-xylosidase were utilized to hydrolyze birchwood xylan. It was interesting to note that endo-xylanase alone produced different concentrations of oligosaccharides (xylobiose, xylotriose and xylotetraose), but after addition of β-xylosidase, most of these xylo-oligosaccharides were converted to xylose (Figure 7). Xylan conversion increased with time. Sugar analysis results revealed that mixture of endo-xylanase and β-xylosidase worked efficiently and converted approximately 90% of xylan to xylose.

## Discussion

Cellulose and hemicellulose components of lignocellulosic biomass are a renewable source of sugars. The main component of hemicellulose is xylan which can be converted into xylose using an effective enzyme system of endo-xylanase and β-xylosidase. β-xylosidase is known to be the key enzyme for converting xylo-oligosaccharides to xylose. Thermostable β-xylosidases produced from thermophiles are of importance due to their prolonged activity during the hydrolysis which may allow a reduced dose of enzyme and cost-efficient conversion [17]. Out of various GH families for β-xylosidase, reports of GH39 family thermostable β-xylosidases are scarce. To date, most of the reported β-xylosidases from *Geobacillus* spp. are from GH52 and GH43 family. For example, β-xylosidases of *Geobacillus thermodenitrificans* TSAA1 [7], *Geobacillus stearothermophilus* [18]*, Geobacillus pallidus* [19], *Bacillus stearothermophilus* 21 [20] belonged to GH52 family and *Geobacillus thermoleovorans* IT-08 [21], *Geobacillus thermoleovorans* IT-08 [22] belonged to GH43 family (Table 2).

**Table 2:** **Properties of thermostable β-xylosidases from thermophiles**

**Organism**	**GH family**	**Optimum pH**	**Optimum temperature (°C)**	**Thermal stability**	**K**_**m**_ **(mM)**	**V**_**max**_ **(U/mg)**	**References**
*Geobacillus* sp. WSUCF1	39	6.5	70	Half-life of 9 days at 70°C	2.3	147	This study
*Thermoanaerobacterium saccharolyticum* JW/SL-YS485	Novel	6.0	65	Half-life of 1 h at 67°C	28.0	276	[5]
*Geobacillus thermodenitrificans* TSAA1	52	7.0	60	Half-life of 3 h at 70°C	2.8	4.16	[7]
*Geobacillus stearothermophilus*	52	6.5	70	Half-life of 3.4 h at 70°C	0.15	NA	[18]
*Bacillus stearothermophilus*	NA	6.0	70	Half-life of approx. 1 h at 70°C	1.2	NA	[10]
*Bacillus thermantarcticus*	NA	6.0	70	Stable for 1 h at 60°C	0.5	NA	[23]
*Geobacillus pallidus*	52	8.0	70	Half-life of approx. 10 h at 70°C	2.4	4.72	[19]
*Geobacillus thermoleovorans* IT-08	43	6.0	60	Retained more than 70 % activity after 1 h at 70°C	0.06	NA	[21]
*Geobacillus thermoleovorans* IT-08	43	5.0	NA	Half-life of 35 min at 57.5°C	0.55	NA	[22]

A high specific activity of β-xylosidase enzyme for xylo-oligosaccharides is an important attribute for efficient enzymatic hydrolysis of the hemicellulose component of lignocellulose [2]. WSUCF1 β-xylosidase specific activity is 133.0 U/mg, which is high in comparison to other bacterial β-xylosidases such as *Thermoanaerobacterium saccharolyticum* JW/SL-YS485 [5], *Geobacillus thermoleovorans* IT-08 [21], *G. thermodenitrificans* TSAA1 [7] and *G. pallidus* [19] that showed specific activity of 45.8, 48.4, 68 and 4.75 U/mg respectively. Specific activity was also higher than the β-xylosidases reported from fungal sources *Paecilomyces thermophila -* 43.4 U mg^−1^ [6]; *Aspergillus awamori* X-100 - 4.2 U mg^−1^ [24] and commercial enzymes like CAZyme™ Xylosidase 1 from Lucigen - 31 U mg^−1^.

Omitting heat denaturation prior to SDS-PAGE resulted in an increase in the apparent mass of the β-xylosidase from 58 kDa to 230 kDa. Interestingly, only the 230 kDa protein displayed xylosidase activity in the zymogram. This suggests that the WSUCF1 β-xylosidase is resistant to full denaturation by the detergent alone, and retains at least some of its quaternary structure which is necessary for successful recovery of catalytic activity following SDS-PAGE. Based on the 4-fold increase in apparent mass, it appears that that the WSUCF1 β-xylosidase exists in tetrameric form. GH39 β-xylosidase from *Thermoanaerobacterium* sp. has been reported to exist as a tetramer [1,25]. Property of retaining its activity in the presence of detergent could make it suitable for commercial applications in need for xylosidases resistant to denaturation with detergents.

WSUCF1 β-xylosidase was active across a broad pH range of 4.5-9.5 with maximum activity at pH 6.5. β-xylosidase from *Geobacillus stearothermophilus* T-6 also exhibited maximum activity at 6.5 [26] whereas β-xylosidases from *Geobacillus thermoleovorans* IT-08 [21], *Geobacillus thermodenitrificans* TSAA1 [7] and *G. pallidus* [19] showed their pH optima at 6.0, 7.0 and 8.0, respectively. Activity at broad pH range is a critical feature of applicability of industrial enzymes. β-xylosidases from other reported *Geobacillus* spp. had a narrow pH range except *G. pallidus* [19]. At pH 9.0, less than 10% and 40% relative activity for *Geobacillus thermoleovorans* IT-08 [21] and *Geobacillus thermodenitrificans* TSAA1 [7], respectively compared to 46% from WSUCF1 β-xylosidase whereas *G. pallidus* [19] retained 89% activity.

WSUCF1 β-xylosidase was also active across a broad temperature range with optima at 70°C. Temperature optima was higher than *Geobacillus stearothermophilus* T-6 (65°C, [26]), *Geobacillus thermoleovorans* IT-08 (65°C, [21]), *Geobacillus thermodenitrificans* TSAA1 (60°C, [7]), and *Thermoanaerobacterium saccharolyticum* JW/SL-YS485 (65°C, [5]). At a temperature higher than its optima (i.e. at 75°C), WSUCF1 β-xylosidase retained 77% relative activity which was comparable to relative activities from *Geobacillus thermodenitrificans* TSAA1 [7] and *G. pallidus* [19]. *Geobacillus thermoleovorans* IT-08 [21] β-xylosidase retained less than 40% relative activity at 75°C.

The performance of an enzyme mainly depends upon its operational stability. Enzymatic hydrolysis of lignocellulosic biomass is usually carried out for ≥ 3 days, so there is utmost requirement of enzymes which could retain their stability during this period. Therefore, thermostable enzymes are gaining wide industrial and biotechnological interest due to their ability to withstand the often relatively harsh conditions of industrial processing [27]. WSUCF1 β-xylosidase was found to be highly thermostable when compared with other reported β-xylosidases from thermophiles. WSUCF1 xylosidase retained 93, 83 and 50% activity at 50, 60 and 70°C after the period of 9 days. β-xylosidase from *Geobacillus pallidus* [19] had a half-life of 50 h at 60°C, *Bacillus halodurans* XylBH39 and XylBH43 displayed approximate half-life values of 2.40 and 0.05 h, respectively at 60°C, *Geobacillus thermoleovorans* IT-08 [21] retained more than 70% activity after 1 h, *Thermoanaerobacterium saccharolyticum* JW/SL-YS485 [5] exhibited half-life of 1 h at 67°C, *Geobacillus thermoleovorans* IT-08 [22] retained 50% activity after 35 min at 57.5°C. At 50°C, the WSUCF1 β-xylosidase retained almost 100% relative activity, and therefore this enzyme could be used as a component of commercial enzyme cocktails mainly working optimally at ≤ 50°C. Various studies have also been published to enhance thermal stability of xylanases using genetic engineering [28,29] whereas WSUCF1 β-xylosidase is highly thermostable in its native form. Thus β-xylosidase from *Geobacillus* sp. WSUCF1 appears to be among the most thermostable β-xylosidases from thermophiles having optimum growth temperatures of ≤ 70°C reported to date (Table 2). Higher thermal-stability of WSUCF1 β-xylosidase will allow hydrolysis for extended times to convert lignocellulosic biomass into sugars, leading to decreased amount of enzyme needed for saccharification. It has also been stated that enzymes are the main cost in lignocellulosic conversion process [30]. Due to high thermostability of WSUCF1 β-xylosidase, it could be recycled with its full activity for the subsequent enzymatic hydrolysis. Several studies have been reported on enzyme recycling, which significantly reduce overall cost of the process [31,32]. These discussions showed that thermostability is an essential attribute of an enzyme for its commercial application.

The kinetic properties of WSUCF1 β-xylosidase (K_m_ - 2.38 mM and V_max_ - 147.0 U/mg) were significantly different from those of the other reported β-xylosidases. Compared to WSUCF1 β-xylosidase, *Thermoanaerobacterium saccharolyticum* JW/SL-YS485 β-xylosidase [5] and *Geobacillus thermodenitrificans* TSAA1 β-xylosidase [7] showed higher K_m_ of 28 mM and 2.8 mM, respectively, which shows WSUCF1 β-xylosidase has more specificity towards substrate.

β-xylosidase plays a vital role in the conversion of xylobiose and other higher xylo-oligosaccharides to xylose during enzymatic hydrolysis [1]. At commercial scale, performing enzymatic hydrolysis of lignocellulosic biomass at high solid loadings is beneficial as it increases product concentrations and utilize less water. On the other end, this increased product concentrations could lead to enzyme inhibition called end-product inhibition [33]. Therefore for industrial processes, utilization of a xylose tolerant β-xylosidase is very important in order to achieve high concentrations of xylose by avoiding end product inhibition. WSUCF1 β-xylosidase exhibited tolerance to high concentrations of xylose, retaining 80% of initial activity at 100 mM xylose i.e. higher tolerance than β-xylosidase from *Paecilomyces thermophila* which retained only 54% activity in the presence of 100 mM xylose. The other β-xylosidases reported for exhibiting strong tolerance to xylose inhibition are from *Scytalidium thermophilum* ([34] and *Thermatoga thermarum* [11] which were insensitive to inhibition by up to 200 mM xylose.

Complete enzymatic degradation of xylan to xylose is one of the most important and challenging reactions. Endoxylanases are reported to deconstruct the xylan into xylose and different xylo-oligosaccharides. These xylo-oligosaccharides are often found in hydrolysates of lignocellulosic materials as they can’t be fermented by ethanalogens leading to lower yields of ethanol [35]. Kont et al. [36] demonstrated that xylo-oligosaccharides are also one of the strong inhibitors of cellulases. Zhang and Viikari [37] and Qing et al. [38] also studied the inhibitory effects of xylo-oligosaccharides on cellobiohydrolase. These studies demonstrate the necessity of enzymes which could breakdown these inhibitory xylo-oligosaccharides to obtain better fermentable sugar yields. Xylo-oligosaccharides are reported to be hydrolyzed to xylose by exo-type xylanolytic β-xylosidase [39]. WSUCF1 β-xylosidase released xylose from all the tested xylo-oligosaccharides (X2-X5) indicating that it is a true β-D-xylosidase [34]. Addition of thermostable WSUCF1 β-xylosidase along with endo-xylanase during birchwood xylan hydrolysis increased final xylose yields to approximately 90% which would further improve the fermentation product yields. Similar observations were observed in β-xylosidases from *Paecilomyces thermophila* [6] and *Thermotoga thermarum* [11], which were active on different xylo-oligosaccharides obtained after xylan hydrolysis.

## Conclusions

In this study, a GH39 β-xylosidase from the thermophile *Geobacillus* sp. strain WSUCF1 was cloned and purified. Compared to β-xylosidases from other thermophiles, WSUCF1 β-xylosidase showed more thermostability. It also displayed various other desirable properties including high specific activity, high xylose tolerance, excellent hydrolytic activity on p-nitrophenyl-xylopyranoside and xylo-oligosaccharides, and resistance to detergent. A high degree of synergy was found with respect to the release of reducing sugars from xylans when β-xylosidase was combined with the endo-xylanase. These findings open a path to potential industrial applications of the WSUCF1 β-xylosidase.

## Methods

### Multiple sequence alignment and phylogenetic analysis

Nucleotide sequence from the whole genome of *Geobacillus* sp. strain WSUCF1 [15] was translated to amino acid sequence using ExPASy-Translate tool (http://web.expasy.org/translate/). Amino acid sequence of *Geobacillus* sp. strain WSUCF1 β-xylosidase was used as a BLAST query for seeking other homologous amino acid sequences. The sequence alignment was created with ClustalW program. Sequences were aligned manually using the Mega 5.2 software. Phylogenetic relationship was inferred using the Neighbor-Joining (NJ) method as described earlier [16].

### Cloning and expression of WSUCF1 β-xylosidase

To amplify the 1509 bp β-xylosidase gene sequence from *Geobacillus* sp. strain WSUCF1 (simply referred to as WSUCF1), PCR primers (F 5’- *CGCGAACAGATTGGAGGT* AAGGTTGTAAACGTGCCAAGC-3’ and R 5’- *GTGGCGGCCGCTCTATTA*TGAAGAATA CGATGTCATTTC-3’) containing homologous sequences to the ends of the pRham N-His SUMO Kan Vector (Lucigen, Middleton, WI) (italicized) were designed to amplify the β-xylosidase gene sequence. PCR product containing the gene of interest is cloned as a fusion to cleavable SUMO (Small Ubiquitin-like Modifier) tag under the control of the L-rhamnose-inducible rhaPBAD promoter harbored on the pRham™ N-His SUMO Vector. PCR conditions were as follows: initial denaturation at 94°C for 2 min followed by 25 cycles of 94°C for 15 sec, 55°C for 15 sec and 72°C for 1 min 30 sec in 50 μl reaction with final extension of 10 min at 72°C. The amplicon was cloned into the vector according to the manufacturer’s instructions, and the resulting plasmid transformed into *E. coli* chemically competent cells (Lucigen, Middleton, WI). After transformation, *E. coli* cells were plated on agar plates containing Terrific broth (TB) and 30 μg/ml kanamycin, incubated for 36 hours at 37°C. The positive clones were screened using colony PCR and confirmed by β-xylosidase activity assay as described below. Cycling conditions for colony PCR were: initial denaturation 94°C, 5 sec; 25 cycles of 94°C for 15 sec, 55°C for 15 sec and 72°C for 1 min; extension 72°C 10 min.

### Purification of the recombinant β-xylosidase

A positive clone was grown in 250-mL flask containing 50 ml Terrific broth (TB), kanamycin (30 μg/ml), and the expression of β-xylosidase was induced by addition of 0.2% rhamnose followed by incubation at 37°C for 48 hours. Cells were centrifuged at 10,000 rpm for 15 minutes. Ten ml of clear culture supernatant was applied to a Ni-NTA agarose resin column (1.5 cm × 5 cm) equilibrated with equilibration buffer (50 mM NaH_2_PO_4_, 300 mM NaCl, 10 mM imidazole, pH 8.0). After binding, the column was washed with wash buffer (50 mM NaH_2_PO_4_, 300 mM NaCl, 20 mM imidazole, pH 8). The bound enzyme was eluted with elution buffer (50 mM NaH_2_PO_4_, 300 mM NaCl, 250 mM imidazole, pH 8.0) at a flow rate of 0.5 ml/min collecting 1 ml fractions. Fractions containing β-xylosidase activity were pooled. To remove the His tag from the protein, the protein was cleaved with SUMO specific protease which also contains the His tag (as per manufacturer’s protocol). After the cleavage, the protein was separated from the SUMO protease and the His tag by re-performing metal affinity chromatography with Ni-NTA agarose resin column. His tag and SUMO protease bound to the resin while the protein was eluted. The homogeneity of the purified enzyme was checked by sodium dodecyl sulfate-polyacrylamide gel electrophoresis (SDS-PAGE) as described below. Protein concentrations in the samples were determined using 2-D Quant kit (GE Healthcare).

### Assay of β-xylosidase activity

For β-xylosidase activity, pNPX, 2.5 mg/mL was used as a substrate. The assay mixture contained 80 μL of substrate, 100 μl 0.1 M phosphate buffer (pH 6.5), and 20 μL of appropriately diluted enzyme. The reaction was carried out at 70°C for 10 min and then stopped by the addition of 100 μL of sodium carbonate (2% w/v). The p-nitrophenol released was determined by measuring the absorbance of each sample at 410 nm. One unit of enzyme activity was defined as the amount of enzyme that released 1 μmol of p-NP per minute under the standard assay conditions.

### SDS-PAGE, zymography, and molecular mass determination

SDS-PAGE was performed as described by Laemmli (1970) [40]. The molecular weight standards used were BioRad Precision plus standards. To obtain zymogram of β-xylosidase activity, enzyme (10 μl) was mixed with 10 μl 2X SDS sample buffer. Two different processing conditions were used prior to loading: i) purified enzyme sample was heated to 95°C for 2 min and then loaded to an 8-16% gradient SDS-PAGE gel and ii) enzyme sample was loaded directly to an 8-16% gradient SDS-PAGE gel without any heating. Current was passed through the gel (150 V constant voltage) for about 1 h until the bromophenol blue dye-front migrated to the bottom of the gel. The gel was washed successively with the following for 30 minutes each: 20% isopropanol in phosphate buffer saline (PBS, pH 5.9); 8 M urea in PBS; and with PBS (pH 5.9) 3 times. The gel was incubated with substrate (0.1 mg/ml 4-methylumbelliferyl-β-D-xylopyranoside in PBS, pH 5.9) for 30 min. Finally, the detection of β-xylosidase activity was examined by fluorescence of 4-methylumbelliferone, which is visualized under ultraviolet light. The gel was then stained with Coomassie brilliant blue R-250 (LabSafe GEL Blue, G-Biosciences, St. Louis, MO) to visualize protein.

### Characterization of WSUCF1 β-xylosidase

Activity profile of β-xylosidase was determined at different pH 3–10. The enzyme assays were carried in the following discontinuous buffer system under standard assay conditions: 100 mM sodium citrate (pH 3–6), sodium phosphate (pH 6–8), Tris–HCl (pH 7.5-9), or glycine-NaOH (pH 8.5-10). The optimum pH was used to determine the optimum temperature for the β-xylosidase. Experiments were carried out in the temperature range of 40-80°C. In order to assess thermostability of β-xylosidase, 1 ml of enzyme was incubated at 50-80°C for varying time intervals. Subsamples (20 μl) were removed at specific time intervals over the period of incubation. The residual enzyme activity was measured and plotted against time. Initial activity was assumed to be 100% and residual enzyme activity reported as percentages of the initial activity during the incubation period.

Kinetic parameters (K_m_ and V_max_) for the purified recombinant β-xylosidase were determined by measuring the enzyme activity using 0–15 mM pNP-X as substrate in 50 mM phosphate buffer (pH 6.5) at 70°C. The data was plotted according to the Lineweaver-Burk method to calculate K_m_ and V_max_ values.

Substrate specificity of the purified enzyme was investigated under standard assay conditions as described above. Activities towards p-nitrophenyl derivatives were measured by the rate of p-nitrophenol formed during hydrolysis from 2.5 mg/ml of the substrates in 50 mM phosphate buffer (pH 6.5) at 70°C for 10 min, and detected by spectrophotometry at 410 nm. Substrates used were p-nitrophenyl β-D-xylopyranoside, p-nitrophenyl α-L-arabinofuranoside, p-nitrophenyl β-D-cellobioside, p-nitrophenyl β-D-glucopyranoside.

The effect of metal ions on enzyme activity of β-xylosidase was determined by adding chloride salts of Mg^2+^, Zn^2+^, Ba^2+^, Ni^2+^, Mn^2+^,Ca^2+^, Co^2+^, Cu^2+^ and Al^3+^ to the enzyme reaction mixtures at a final concentration of 1 mM. The enzyme reaction mixtures were incubated for 10 min at 70°C, and then activities were measured as described above. The activity of the enzyme without adding any metal ions was considered as 100%.

The inhibitory effect of different concentrations of xylose was determined by incubating 20 μl enzyme solution, 80 μl of 2.5 mg/ml pNPX dissolved in 100 mM phosphate buffer (pH 6.5) and varying amounts of xylose with a final concentration of 0–300 mM at 70°C for 10 min. Residual β-xylosidase activities were measured as described above.

### Hydrolysis of xylo-oligosaccharides by the WSUCF1 β-xylosidase

Four μl of 1.5% (w/v) xylo-oligosaccharides was incubated with 0.05 U of the β-xylosidase in 50 mM phosphate buffer (pH 6.5) at 70°C for 1, 2 and 6 h. Aliquots were withdrawn at 0, 1, and 2 h, the reactions were stopped by boiling for 5 min. Products of enzymatic hydrolyses were analyzed by spotting on silica gel plates 60 F-254 (E. Merck, Germany). The plates were developed with butanol-acetic acid-water (2:1:1, v/v/v) solvent system followed by heating for few minutes at 105°C in an oven, after spraying the plates with a methanol-sulfuric acid mixture (9:1, v/v) [11]. A mixture of xylo-oligosaccharides consisting of xylose (X), xylobiose (XB), xylotriose (XTri), and xylotetraose (XTet) and xylopentaose (XP) were used as standards.

### Synergistic effect of WSUCF1 endo-xylanase and β-xylosidase on xylan hydrolysis

For enzymatic hydrolysis of xylan, the reaction mixture containing 5 mg purified recombinant endoxylanase/g xylan (purified enzyme from *Geobacillus* sp. WSUCF1, [16]), 2.5 mg recombinant β-xylosidase/g xylan and 20 mg birchwood xylan in 4 ml of 50 mM phosphate buffer (pH 6.5) was incubated at 60°C for 6 to 24 h. Xylose, xylobiose, xylotriose and xylotetraose were determined by high performance liquid chromatography using a 30 mm Micro-Guard Cation H cartridge and a 300 mm Aminex HPX-87H column (Bio-Rad Laboratories, Inc., Hercules, CA) on a 1100 Series HPLC system equipped with a refractive index detector (Agilent Technologies, Santa Clara, CA). Samples (10 μl) were injected onto a heated column (55°C) and eluted at 0.6 ml/min using 5 mM H_2_SO_4_ as mobile phase.
